# Oxygen-Sensing Nox4 Generates Genotoxic ROS to Induce Premature Senescence of Nucleus Pulposus Cells through MAPK and NF-*κ*B Pathways

**DOI:** 10.1155/2017/7426458

**Published:** 2017-09-24

**Authors:** Chencheng Feng, Yang Zhang, Minghui Yang, Minghong Lan, Huan Liu, Bo Huang, Yue Zhou

**Affiliations:** Department of Orthopedics, Xinqiao Hospital, Third Military Medical University, Chongqing 400037, China

## Abstract

Senescence is a crucial driver of intervertebral disc degeneration (IDD). Disc cells are exposed to high oxygen tension due to neovascularization in degenerative discs. However, the effect of oxygen tension on disc cell senescence was unknown. Herein, rat nucleus pulposus (NP) cells were cultured under 20% O_2_ or 1% O_2_. Consequently, ROS induced by 20% O_2_ caused DNA damage and then activated p53-p21-Rb and p16-Rb pathways via ERK signaling to induce NP cell senescence. It also induced catabolic and proinflammatory phenotype of NP cells via MAPK and NF-*κ*B pathways. Furthermore, 20% O_2_ was found to upregulate Nox4 in NP cells. Small interfering RNA against Nox4 reduced ROS production induced by 20% O_2_ and consequently suppressed premature senescence of NP cells. On the contrary, NP cells overexpressing Nox4 produced more ROS and rapidly developed senescent signs. In consistent with the in vitro studies, the expression of Nox4, p21, and Rb was upregulated in rat degenerative discs. This study, for the first time, demonstrates that Nox4 is an oxygen-sensing enzyme and a main ROS source in NP cells. Nox4-dependent ROS are genotoxic and a potent trigger of NP cell senescence. Nox4 is a potential therapeutic target for disc cell senescence and IDD.

## 1. Introduction

The accumulation of senescent intervertebral disc (IVD) cells is a hallmark of IVD degeneration (IDD) [1, 2]. Senescent disc cells are irreversibly cell cycle arrested, causing a decrease in the number of functional and viable disc cells gradually due to apoptosis or necrosis. On the other hand, an aberrant secretion of extracellular matrix (ECM) catabolic proteases and proinflammatory mediators is strongly associated with the senescence-associated secretory phenotype (SASP) of disc cells [3, 4]. The SASP causes ECM turnover and inflammation in the microenvironment of IVDs and also exerts negative effects on neighboring normal disc cells [5, 6]. Together, disc cell senescence is crucial to the establishment and progression of IDD [7, 8]. Investigating the molecular mechanism underlying disc cell senescence contributes to understanding the pathogenesis of IDD further in depth. Preventing or delaying disc cell senescence is a potential therapeutic approach for IDD.

The microenvironment of degenerative discs is characterized by acidic PH, low nutrition, high osmotic pressure, and inflammation [9]. Oxygen tension in avascular IVDs is low (1% O_2_ in nucleus pulposus (NP)). NP cells are subjected to a hypoxic but not entirely anaerobic microenvironment [10, 11]. NP cells adapt to hypoxic microenvironment well and still have a certain level of oxidative metabolism [12, 13]. Nevertheless, degenerative discs with neovascularization are characterized by increased oxygen tension. Therefore, the resident NP cells are exposed to higher oxygen tension [14, 15]. High oxygen tension (20% O_2_) has been reported to enhance the production of mitochondrial-derived reactive oxygen species (ROS) in NP cells to perturb ECM homeostasis of IVDs [16]. On the contrary, lower oxygen tension (2% O_2_) was shown to decrease ROS production in NP cells and consequently to suppress the excessive autophagy of NP cells [17]. The findings suggest that oxygen tension affects the function and viability of NP cells through regulating ROS metabolism. However, the effect of oxygen tension on disc cell senescence remains unknown.

Numerous lines of evidence indicate that ROS are involved in the signal transduction, ECM metabolism, programed cell death, senescence, and phenotypic shift of disc cells [4, 18, 19]. With respect to the source of ROS in disc cells, mitochondrion is a major site of ROS generation in disc cells [16, 20]. However, nonmitochondrial ROS production through nicotinamide adenine dinucleotide phosphate (NADPH) oxidase (Nox) in disc cells is unclear. The Nox family consists of seven isoforms (Nox1–5 and dual oxidase 1-2). They are professional ROS-generating enzymes and have been documented in various diseases, including cardiovascular diseases, lung injury, and central nervous system diseases [21–23]. Uniquely, Nox4 is an oxygen-sensing and constitutively active enzyme [24, 25]. It is abundant in kidneys, vascular system, and many other tissues [26, 27], suggesting a more general function of Nox4 than that of some other Nox members. Nox4-depedent ROS have been identified as a critical mediator in cell senescence induced by resveratrol, oncogene, and cytokine [28–30]. However, the expression of Nox4 in disc cells has not been determined. The role of Nox4 in disc cell senescence is unclear.

In this study, we investigated the effect of oxygen tension in 20% O_2_ or 1% O_2_ on disc cell senescence using rat NP cells. And then, the involvement of Nox4-dependent ROS in mediating the regulatory effect of oxygen tension on NP cell senescence was analyzed. At the same time, the association between Nox4 and IDD was investigated using the rat caudal IDD model produced by disc needle puncture. Furthermore, the role of MAPK and NF-*κ*B pathways on the downstream of ROS in regulating NP cell senescence was also evaluated. Our study gives a novel insight into the mechanism of disc cell senescence and the pathogenesis of IDD. The abbreviations used in this study are listed in the supplementary materials (Supplementary Material, Table 1 available online at https://doi.org/10.1155/2017/7426458).

## 2. Materials and Methods

### 2.1. Ethics Statement

This research was approved by the Ethical Committee of Xinqiao Hospital. All procedures described in this study were in accordance with the standards set forth in the eighth edition of Guide for the Care and Use of Laboratory Animals published by the National Academy of Sciences (Washington, DC, USA).

### 2.2. Reagents

Glutathione (GSH) and the NF-*κ*B inhibitor (PDTC) were bought from Beyotime (Shanghai, China). Ν-acetyl-L-cysteine (NAC) and the antibody against BrdU (B8434) were provided by Sigma (St. Louis, MO, USA). The antibodies against p65 (ab16502) and phospho-p65 (p-p65, ab76302) were purchased from Abcam (Cambridge, MA, USA). The antibodies against phosphor-histone *γ*-H2A.X (number 9718), p38 (number 9212), phospho-p38 (p-p38, number 9211), JNK (number 9252), phospho-JNK (p-JNK, number 9251), Erk1/2 (number 9102), and phospho-Erk1/2 (number 9101) were obtained from Cell Signaling Technology (Danvers, MA, USA). The antibodies against GAPDH (sc-47724), p53 (sc-126), p21 (sc-6246), p16 (sc-1661), phosphorylated retinoblastoma protein (p-Rb, sc-377528), and Rb (sc-50) were purchased from Santa Cruz Biotechnology (Santa Cruz, CA, USA). The antibody against Nox4 (NB110-58851) was provided by Novus Biologicals (Littleton, CO, USA). The IgG Alexa Fluor® 647-conjugated secondary antibodies (AP192SA6 and AP187SA6) were purchased from Merck Millipore (Millipore Billerica, MA, USA). The horseradish peroxidase- (HRP-) conjugated secondary antibodies (ZB2305 and ZB2301) were purchased from ZSGB-BIO (Beijing, China). The p38 inhibitor (SB202190, SB), the JNK inhibitor (SP600125, SP), and the ERK inhibitor (U0126, U) were provided by MedChemexpress (Princeton, NJ, USA).

### 2.3. Rat NP Cell Culture at 1% O_2_ and High Oxygen Tension (20% O_2_) Treatment

Rat NP cells were isolated as previously mentioned [31]. Isolated NP cells were cultured at 37°C and 1% O_2_. We cultured rat NP cells in low oxygen tension until they reached to the second or third passages. The period the cells remained in low oxygen tension was 4-5 weeks. To increase oxygen tension, NP cells were treated with 20% O_2_ for 72 h. In order to scavenge ROS, NP cells were preincubated with GSH (1 mg/ml) and NAC (100 *μ*g/ml) for 30 min before high oxygen treatment. Then, DMEM/F12 medium containing GSH or NAC was updated every 24 h. The cells used in this study were at the second or third passages.

### 2.4. Small Interfering RNA (siRNA) against Nox4 (siNox4)

NP cells were transfected with siNox4 (RiboBio, Guangzhou, Guangdong, China) or scrambled siRNA control (siCtrl) using lipofectamine 2000 transfection reagent (Invitrogen, Carlsbad, CA, USA). The siNox4 sequences were 5′- GCAGAGACATCCAATCATT-3′. Next, the transfected NP cells were treated with 20% O_2_ for 72 h. The knockdown efficiency was checked by reversed transcription-quantitative PCR (RT-qPCR) and immunoblot analysis.

### 2.5. Nox4 Overexpression

NP cells at 1% O_2_ or 20% O_2_ were transfected with pRP[EXP]-EGFP-EF1A>rNox4 vector (Cyagen, Santa Clara, CA, USA) using lipofectamine 2000. At 48 h after transfection, the expression of green fluorescent protein (GFP) in the transfected cells was observed under a microscope (Olympus, Tokyo, Japan). The expression of Nox4 was measured using RT-qPCR and immunoblot analysis.

### 2.6. Treatment of MAPK and NF-*κ*B Inhibitors

The phosphorylation of ERK, JNK, p38, and p65 was investigated in NP cells treated with 20% O_2_ for 10, 30, 45, 60, and 120 min using immunoblot analysis. In order to determine the role of Nox4-dependent ROS in the activation of MAPK and NF-*κ*B, NP cells were pretreated with GSH, NAC, and siNox4 as mentioned above followed by high oxygen treatment. Furthermore, NP cells were pretreated with the p38 inhibitor (SB202190, 1 *μ*g/ml), the JNK inhibitor (SP600125, 1 *μ*g/ml), the ERK inhibitor (U0126, 1 *μ*g/ml), and the NF-*κ*B inhibitor (PDTC, 1 *μ*g/ml) for 30 min followed by high oxygen treatment to determine the roles of MAPK and NF-*κ*B pathways in regulating NP cell senescence.

### 2.7. SA-*β*-gal Staining

The SA-*β*-gal staining of NP cells was investigated using SA-*β*-gal staining kit (Cell Signaling Technology). Fixative NP cells seeded in 12-well culture plates were incubated with the staining solution at 37°C for 12 h. Nine random fields per well were imaged using a phase-contrast microscope (200x magnification, Olympus). The mean percentage of SA-*β*-gal-positive cells was calculated.

### 2.8. ROS Measurement

ROS levels in NP cells were analyzed using the Amplex Red Hydrogen Peroxide/Peroxidase Assay kit (Thermo Scientific, Waltham, MA, USA). 20 *μ*l of NP cell suspension (1-1.5 × 10^4^) was incubated with 100 *μ*l of Amplex Red reaction mixture in 96-well microplate at 37°C for 10 min. The absorbance was measured at 560 nm using a spectrophotometer (Varioskan Flash, Thermo Scientific, Waltham, MA, USA). The concentration of ROS was calculated based on the absorbance according to the H_2_O_2_ standard curve.

### 2.9. BrdU Incorporation

For BrdU incorporation, NP cells seeded in coverslips were incubated with DMEM/F12 medium containing BrdU (1 *μ*g/ml, BD Biosciences, San Jose, CA, USA) for 24 h. After fixation with 4% paraformaldehyde, NP cells were incubated with 1 ml HCl (2 mol/l) under room temperature for 30 min. Then, the cells were used to perform the immunofluorescence assay.

### 2.10. Immunofluorescence Assay

The NP cells with BrdU incorporation were used to perform BrdU staining. For *γ*-H2A.X staining, NP cells seeded in coverslips were fixed with 4% paraformaldehyde. After permeabilization and antigen retrieval, NP cells were incubated with the primary antibodies against BrdU (1 : 500 dilution) and *γ*-H2A.X (1 : 400 dilution) overnight at 4°C. After rinsing, NP cells were incubated with the Alexa Fluor 647 dye-conjugated secondary antibodies (1 : 400 dilution) in the dark and then stained with DAPI (0.1 mg/ml, Sigma). The cells without primary antibody incubation served as the negative control. Three random fields per coverslip were imaged using a confocal microscope (200x magnification, Leica, Wetzlar, Germany). The mean percentages of BrdU-positive cells and *γ*-H2A.X-positive cells were calculated by using Image J software.

### 2.11. RT-qPCR

Total RNA was isolated from NP cells using TRIzol reagent (Takara Bio, Shiga, Japan) and quantitated using a NanoDrop ND-2000 spectrophotometer (Thermo Scientific, Waltham, MA, USA). 1 *μ*g RNA was reverse transcribed into cDNA using a Prime Script RT reagent kit (Takara Bio). Real-time quantitative PCR was carried out in triplicate using a ViiA™7 Real-Time PCR system (Thermo Scientific). The SYBR® Premix Ex Taq™ II kit (Takara Bio) was used. The reaction condition was 95°C for 30 seconds, followed by 40 cycles of 95°C for 5 seconds and 60°C for 30 seconds. The relative expression of genes was calculated using the 2^−ΔΔCt^ method. GAPDH served as the internal reference gene. The primers used in the current study are listed in Table 1. The expression of matrix metalloproteinase 2 (MMP2), MMP3, MMP13, a disintegrin and metalloproteinase with thrombospondin motifs 4 (ADAMTS4), ADAMTS5, interleukin 6 (IL6), C-C motif ligand 2 (CCL2), CCL5, and C-X-C motif ligand 10 (CXCL10) in NP cells was analyzed to investigate the SASP of rat NP cells.

### 2.12. Immunoblot Analysis

Total proteins of NP cells were extracted using protein extraction reagent (Thermo Scientific) and quantified using BCA kit (Beyotime). Proteins mixed with loading buffer (Invitrogen) were electrophoresed on SDS gels and then were transferred to PVDF membranes (Millipore). The membranes were blocked using 5% milk proteins in TBST for 1 h at 37°C. Next, the membranes were incubated with primary antibodies against GAPDH (1 : 1000 dilution), p53 (1 : 700 dilution), p21 (1 : 500 dilution), Rb (1 : 700 dilution), p-Rb (1 : 700 dilution), p38 (1 : 1000 dilution), p-p38 (1 : 1000 dilution), Erk1/2 (1 : 1000 dilution), p-ERK1/2 (1 : 1000 dilution), JNK (1 : 1000 dilution), p-JNK (1 : 1000 dilution), p65 (1 : 1000 dilution), p-p65 (1 : 1000 dilution), and Nox4 (1 : 100 dilution) overnight at 4°C, followed by incubation with the HRP-conjugated secondary antibodies (1 : 400 dilution) at 37°C for 1 h. Immunolabeling was detected using electrochemiluminescence reagent (Thermo Scientific). The optical density (OD) of blot bands was measured using Image J software (National Institutes of Health, USA). The relative expression fold of each protein = OD of each protein in the treatment group/OD of each protein in the control group. The adjusted relative expression fold of each protein = the relative expression fold of each protein/the relative expression fold of GAPDH. The phosphorylation level of each protein = the relative expression fold of phosphor-protein/the relative expression fold of total protein.

### 2.13. Rat Caudal IDD Model

A total of ten eight-week-old male SD rats were used. The rats were randomized into two groups. Under anesthesia, caudal discs (C6–C9) were exposed through posterior incision. Disc puncture was performed using a 23-gauge needle on C7-C8 to cause IDD, as mentioned previously [19, 32, 33]. Untreated C6-C7 and C8-C9 served as the internal control. For the sham-operated control, caudal discs were exposed without puncture. After surgery, the rats were fed normally and monitored. MRI was performed 1 month after surgery. Caudal discs were harvested for H&E staining and immunohistochemistry analysis.

### 2.14. Immunohistochemistry Analysis

Deparaffinized IVD sections were incubated with primary antibodies against Nox4 (1 : 10 dilution), p21 (1 : 50 dilution), and Rb (1 : 50 dilution) overnight at 4°C followed by the incubation with HRP-conjugated secondary antibodies at room temperature. Nuclei were counterstained with hematoxylin. All sections were viewed under a microscope (Olympus). The integrated optical density (IOP) and area of sections were measured using Image-Pro Plus 6.0. The mean optical density (MOD, MOD = IOP/area) of sections was calculated to determine the expression intensity of targeted proteins.

### 2.15. Statistical Analysis

All experiments were performed independently in three replicates at least. The results are presented as mean ± SEM. For comparisons between two independent groups, a two-tailed Student's *t*-test was used. Comparisons between three or more groups were tested by one-way ANOVA and LSD multiple comparisons. The results of RT-qPCR analysis and immunoblot analysis were statistically tested using Kruskal-Wallis nonparametric analysis and Mann–Whitney *U* post hoc tests. All results were analyzed using GraphPad Prism 6 and SPSS version 22.0 software programs. *P* < 0.05 was considered statistically significant.

## 3. Results

### 3.1. High Oxygen Tension Caused Excessive ROS Production to Induce NP Cell Senescence

**Figure 1:**
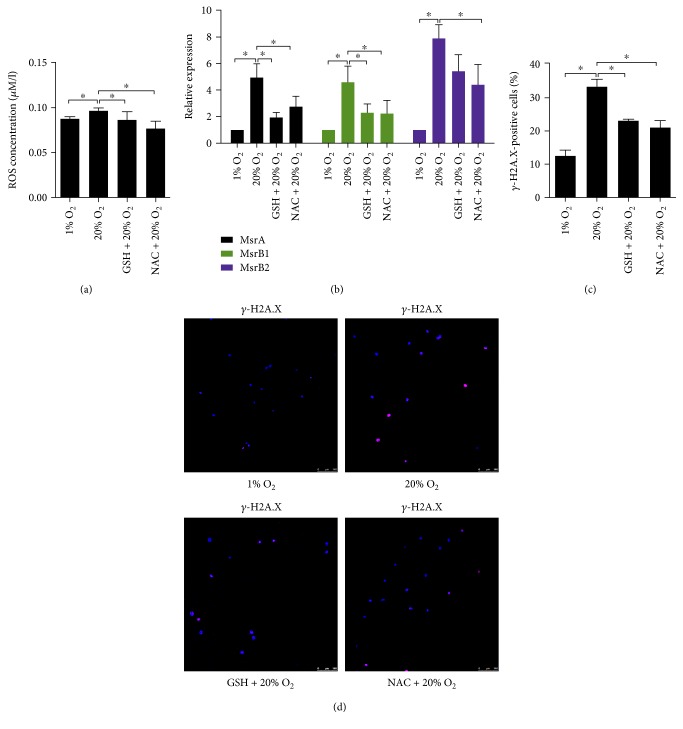
High oxygen tension increased ROS production to induce DNA damage in nucleus pulposus (NP) cells. (a) ROS production in high oxygen tension-treated NP cells (*N* = 3). (b) Quantitative PCR analysis of methionine sulfoxide reductase A (MsrbA), MsrB1, and MsrB2 in high oxygen tension-treated NP cells (*N* = 6). (c, d) Immunofluorescence staining of *γ*-H2A.X and percentage of *γ*-H2A.X-positive cells in high oxygen tension-treated NP cells (*N* = 5). NP cells were pretreated with glutathione (GSH) and *Ν*-acetyl-L-cysteine (NAC) for 30 min followed by high oxygen tension treatment for ROS scavenging. ^∗^*P* value < 0.05, error bars represent standard error.

**Figure 2:**
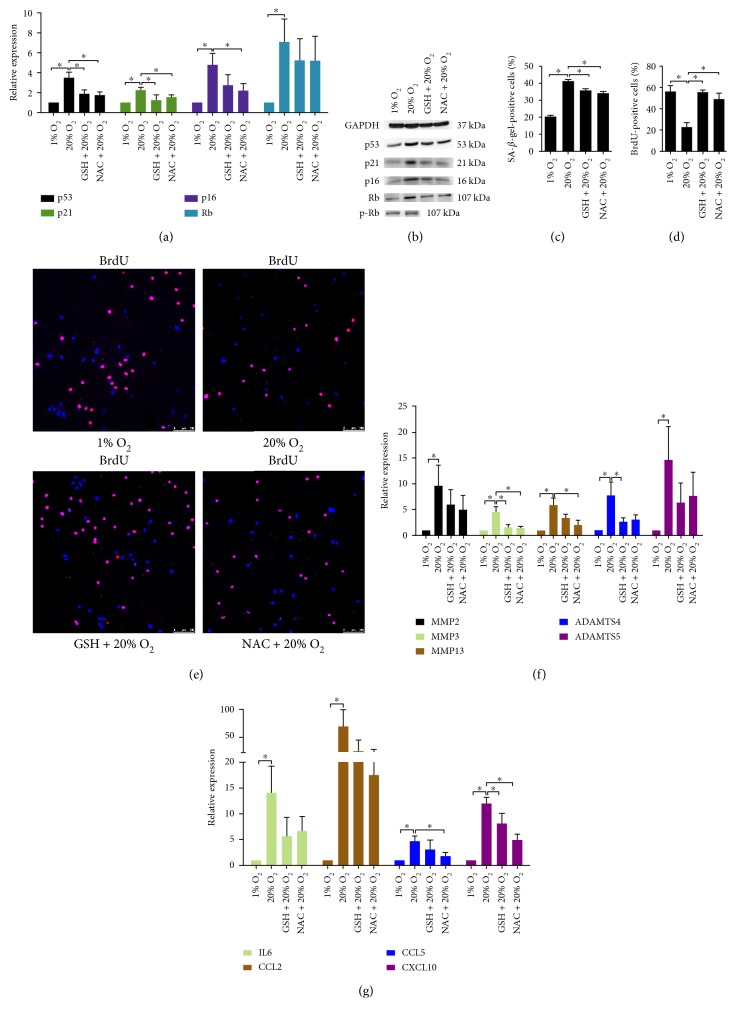
High oxygen tension induced premature senescence of NP cells through ROS/oxidative stress. (a, b) Quantitative PCR analysis (*N* = 4) and representative immunoblot analysis of p53, p16, p21, Rb, and p-Rb in high oxygen tension-treated NP cells. (c) The percentage of SA-*β*-gal-positive NP cells (*N* = 8). (d, e) Immunofluorescence staining of BrdU and percentage of BrdU-positive cells in high oxygen tension-treated NP cells (*N* = 8). (f, g) RT-qPCR analysis of matrix proteases and proinflammatory cytokines in high oxygen tension-treated NP cells (*N* = 5). NP cells were pretreated with GSH and NAC for 30 min followed by high oxygen tension treatment for ROS scavenging. ^∗^, *P* value < 0.05, error bars represent standard error.

20% O_2_ significantly enhanced ROS production in NP cells (Figure 1(a)). At the same time, the expression of methionine sulfoxide reductase A (MsrA), MsrB1, and MsrB2 in NP cells was upregulated by 20% O_2_ (Figure 1(b)). Msr catalyzes the reduction of methionine residues in proteins to repair the oxidative damage of proteins. It has been recognized as an oxidative stress marker of disc cells [34]. High oxygen tension also increased the percentage of *γ*-H2A.X-positive cells, indicating an enhanced DNA damage induced by high oxygen tension (Figures 1(c) and 1(d)). The expression of p53, p21, p16, and Rb in NP cells was significantly upregulated by high oxygen tension (Figures 2(a) and 2(b), Supplementary Material, Figure S7A). However, the protein level of p-Rb was not affected by high oxygen tension (Figure 2(b), Supplementary Material, Figure S7B). Concomitantly, NP cells subjected to 20% O_2_ showed an increased percentage of SA-*β*-gel-staining and a decreased BrdU incorporation compared with those at 1% O_2_ (Figures 2(c), 2(d), and 2(e), Supplementary Material, Figure S1). In addition, the catabolic and proinflammatory signaling in NP cells also was activated by high oxygen tension. The expression of MMP2, MMP3, MMP13, ADAMTS4, ADAMTS5, IL6, CCL2, CCL5, and CXCL10 in NP cells was markedly upregulated (Figures 2(f) and 2(g)). In summary, high oxygen tension induced premature senescence of NP cells.

In order to elucidate the role of ROS in high oxygen tension-induced premature senescence of NP cells, GSH and NAC were used. As a result, both antioxidants suppressed ROS production and expression of MsrA, MsrB1, and MsrB2 in NP cells treated with high oxygen tension (Figures 1(a) and 1(b)). The percentage of *γ*-H2A.X-positive cells was significantly reduced by the antioxidants (Figures 1(c) and 1(d)). Subsequently, NP cell senescence was retarded. Moreover, the inductive effect of high oxygen tension on the catabolic and proinflammatory phenotype of NP cells was antagonized by both antioxidants (Figure 2, Supplementary Material, Figure S1, S7A).

### 3.2. MAPK and NF-*κ*B Pathways Mediated High Oxygen Tension-Induced Premature Senescence of NP Cells

**Figure 3:**
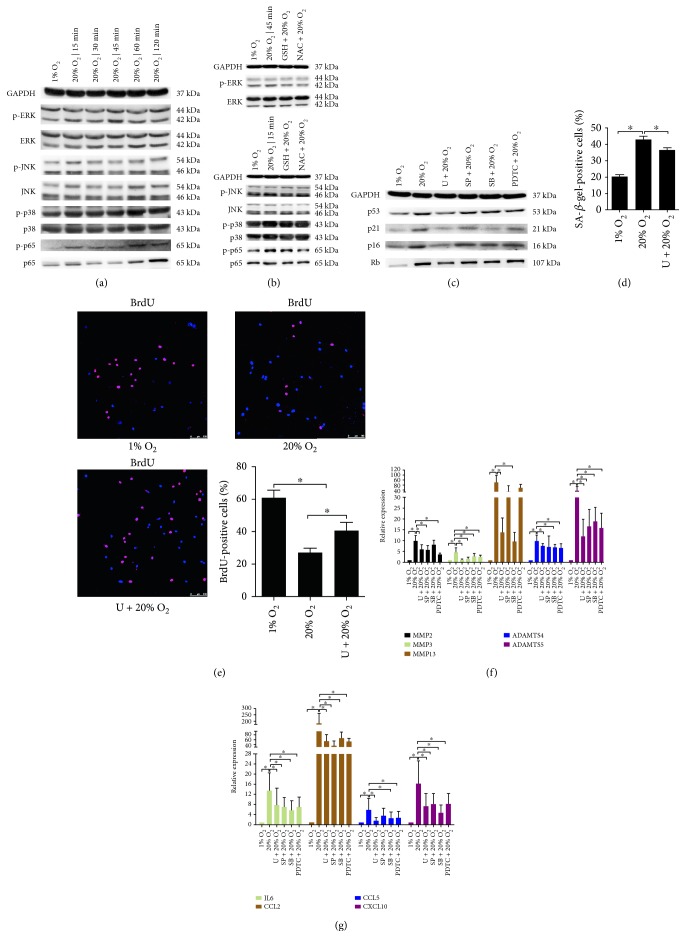
High oxygen tension regulated the cell cycle and senescence-associated secretory phenotype of NP cells via MAPK and NF-*κ*B signaling pathways. (a, b) Representative immunoblot analysis of p38, JNK, p65, and ERK in NP cells. High oxygen tension activated p38, JNK, p65, and ERK in NP cells. The MAPK and NF-*κ*B signaling pathways were the downstream signaling of ROS in NP cells. (c) Representative immunoblot analysis of p53, p16, p21, and retinoblastoma protein (Rb) in NP cells. (d) The percentage of SA-*β*-gal-positive NP cells (*N* = 8). (e) Immunofluorescence staining of BrdU and percentage of BrdU-positive cells in NP cells (*N* = 8). (f, g) RT-qPCR analysis of matrix degradation enzymes and proinflammatory cytokines in NP cells (*N* = 4). NP cells were pretreated with GSH, NAC, the p38 inhibitor (SB202190, SB), the JNK inhibitor (SP600125, SP), the ERK inhibitor (U0126, U), or the NF-*κ*B inhibitor (PDTC) for 30 min followed by high oxygen tension treatment for ROS scavenging or signaling inhibition. ^∗^, *P* value < 0.05, error bars represent standard error.

JNK, p38, and p65 showed an enhanced phosphorylation after high oxygen tension treatment for 15 min while ERK showed an enhanced phosphorylation at 45 min (Figure 3(a), Supplementary Material, Figure S8A). GSH and NAC suppressed the phosphorylation of p38, ERK, JNK, and p65 in NP cells subjected to high oxygen tension (Figure 3(b), Supplementary Material, Figure S8B). On the other hand, U0126 dramatically downregulated the expression of p53, p21, p16, and Rb in NP cells more potently than SP600125, SB202190, and PDTC (Figure 3(c), Supplementary Material, Figure S7E). Consequently, the percentage of SA-*β*-gel-positive cells decreased, and the BrdU incorporation of NP cells increased (Figures 3(d) and 3(e), Supplementary Material, Figure S2). Noticeably, all signaling inhibitors suppressed the high oxygen tension-induced expression of matrix proteases and cytokines in NP cells (Figures 3(f) and 3(g)).

### 3.3. Nox4 Was a Critical Mediator in High Oxygen Tension-Induced Premature Senescence of NP Cells

**Figure 4:**
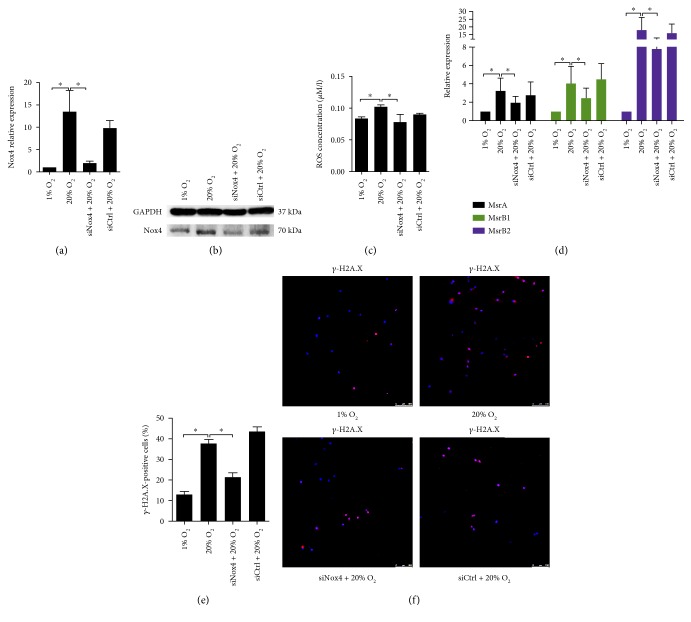
Small interfering RNA against Nox4 (siNox4) alleviated oxidative stress and DNA damage induced by high oxygen tension in NP cells. (a, b) RT-qPCR analysis (*N* = 3) and representative immunoblot analysis of Nox4 in NP cells. The knockdown of Nox4 in NP cells was confirmed. (c) ROS production in NP cells (*N* = 3). (d) RT-qPCR analysis of MsrbA, MsrB1, and MsrB2 in NP cells (*N* = 3). (e, f) Immunofluorescence staining of *γ*-H2A.X and percentage of *γ*-H2A.X-positive cells in NP cells (*N* = 6). NP cells were transfected with siNox4 or scrambled siRNA control (siCtrl) before high oxygen tension treatment. ^∗^, *P* value < 0.05, error bars represent standard error.

**Figure 5:**
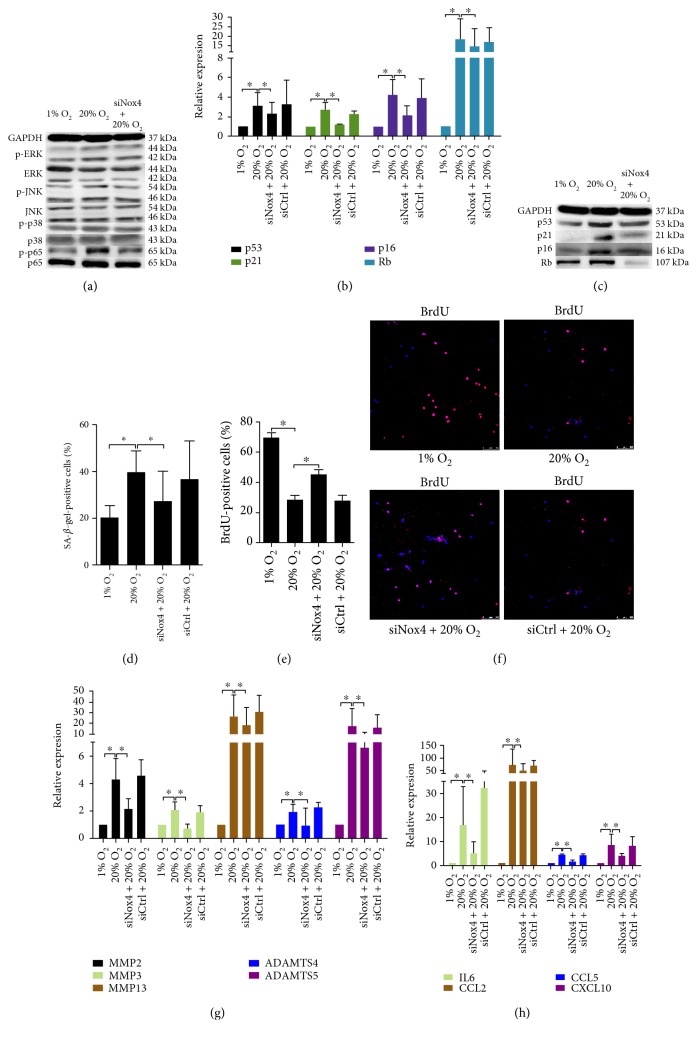
Small interfering RNA against Nox4 (siNox4) retarded high oxygen tension-induced premature senescence of NP cells. (a) Representative immunoblot analysis showed that p38, JNK, ERK, and p65 were on the downstream of Nox4 in NP cells. (b, c) RT-qPCR analysis (*N* = 3) and representative immunoblot analysis of p53, p16, p21, and Rb in NP cells. (d) The percentage of SA-*β*-gal-positive NP cells (*N* = 8). (e, f) Immunofluorescence staining of BrdU and percentage of BrdU-positive cells in NP cells (*N* = 8). (g, h) RT-qPCR analysis of matrix degradation enzymes and proinflammatory cytokines in NP cells (*N* = 3). NP cells were transfected with siNox4 or scrambled siRNA control (siCtrl) before high oxygen tension treatment. ^∗^, *P* value < 0.05, error bars represent standard error.

High oxygen tension-induced Nox4 expression in NP cells was prominently knockdown by siNox4 (Figures 4(a) and 4(b), Supplementary Material, Figure S9A). Consequently, ROS production and Msr expression in NP cells were decreased (Figures 4(c) and 4(d)). The percentage of *γ*-H2A.X-positive cells declined (Figures 4(e) and 4(f)). Moreover, the phosphorylation of p38, ERK, JNK, and p65 in NP cells induced by high oxygen tension was markedly suppressed siNox4. Concomitantly, the expression of p53, p21, p16, and Rb in NP cells was downregulated (Figures 5(a), 5(b), and 5(c), Supplementary Material, Figure S7C, S8C). The decreased BrdU incorporation and increased SA-*β*-gel-staining of NP cells induced by high oxygen tension were abolished (Figures 5(d), 5(e), and 5(f), Supplementary Material, Figure S3) while the catabolic and proinflammatory signaling in NP cells was also inhibited (Figures 5(g) and 5(h)).

### 3.4. Overexpression of Nox4 Enhanced the ROS Production and Accelerated NP Cell Senescence

**Figure 6:**
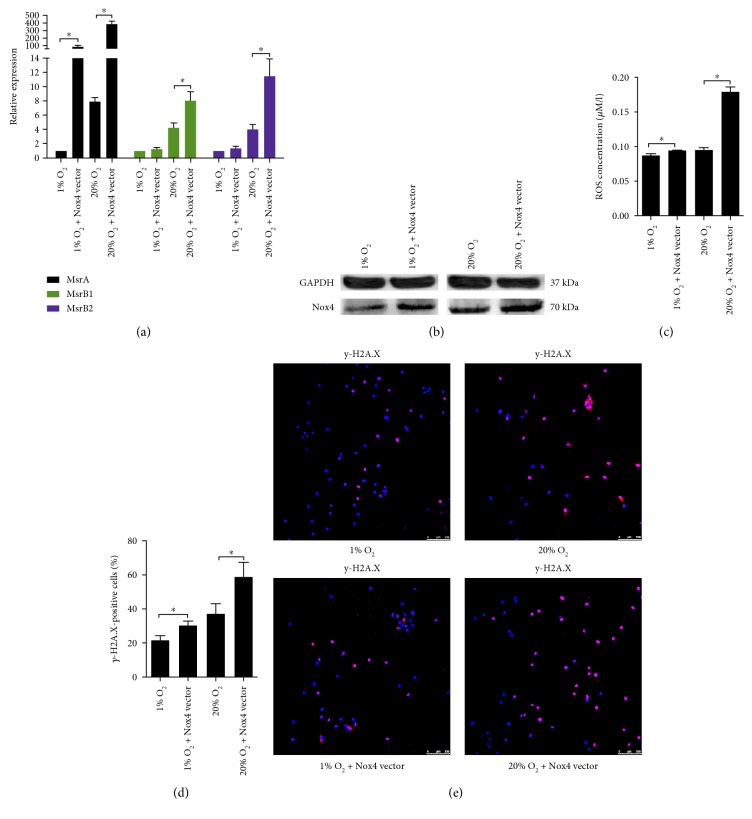
Nox4 overexpression boosted ROS production and induced DNA damage in NP cells. (a) RT-qPCR analysis (*N* = 4) of Nox4, MsrB1, and MsrB2 in NP cells overexpressing Nox4. (b) Representative immunoblot analysis of Nox4 in NP cells overexpressing Nox4. (c) ROS production in NP cells overexpressing Nox4 (*N* = 3). (d, e) Immunofluorescence staining of *γ*-H2A.X and percentage of *γ*-H2A.X-positive cells in NP cells overexpressing Nox4 (*N* = 8). NP cells were transfected with Nox4 vectors for Nox4 overexpression. ^∗^, *P* value < 0.05, error bars represent standard error.

**Figure 7:**
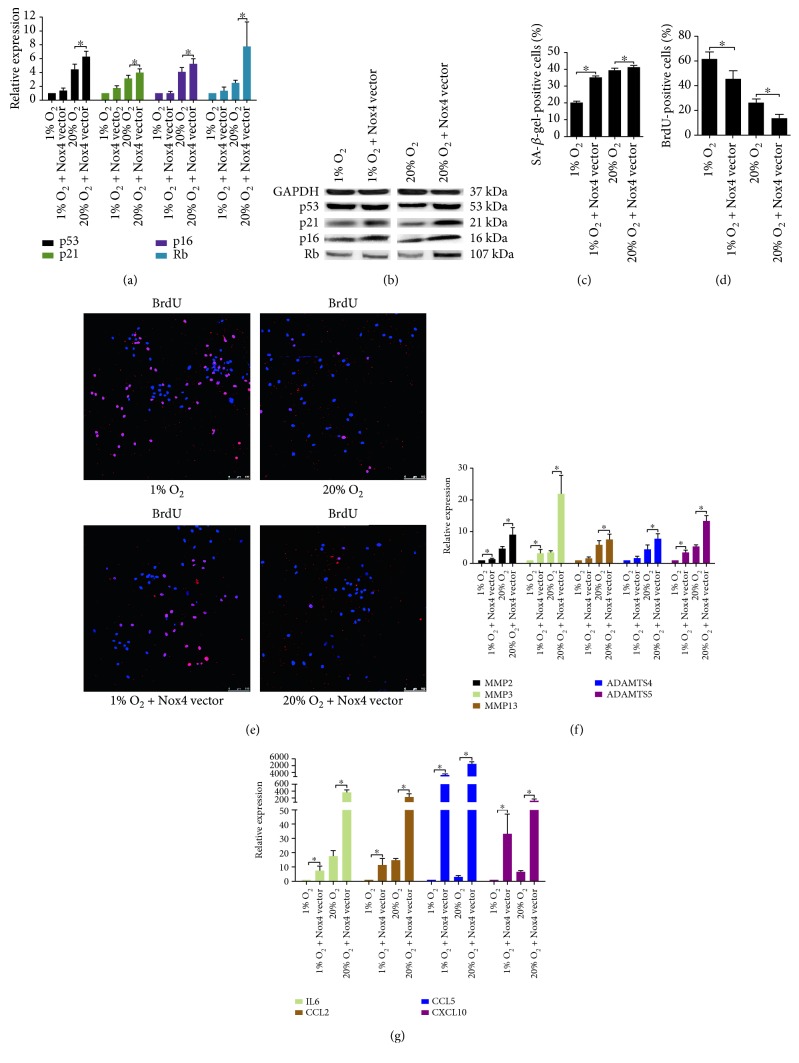
Nox4 overexpression accelerated senescence of NP cells. (a, b) RT-qPCR analysis (*N* = 4) and representative immunoblot analysis of p53, p16, p21, and Rb in NP cells overexpressing Nox4. (c) The percentage of SA-*β*-gal-positive NP cells overexpressing Nox4 (*N* = 8). (d, e) Immunofluorescence staining of BrdU and percentage of BrdU-positive cells in NP cells overexpressing Nox4 (*N* = 8). (f, g) RT-qPCR analysis of matrix degradation enzymes and proinflammatory cytokines in NP cells overexpressing Nox4 (*N* = 4). NP cells were transfected with Nox4 vectors for Nox4 overexpression. ^∗^, *P* value < 0.05, error bars represent standard error.

NP cells transfected with the vectors synthesized GFP (Supplementary Material, Figure S4) and showed an increased Nox4 expression (Figures 6(a) and 6(b), Supplementary Material, Figure S9B). Accordingly, an increased ROS production along with an upregulation of MsrB1 and MsrB2 was observed in the transfected cells (Figures 6(a) and 6(c)). As also, the percentage of *γ*-H2A.X-positive cells increased (Figures 6(d) and 6(e)). The expression of p53, p21, p16, and Rb in the transfected cells was slightly upregulated at 1% O_2_ and was markedly upregulated at 20% O_2_ (Figures 7(a) and 7(b), Supplementary Material, Figure S7D), causing an increased SA-*β*-gel-staining and a decreased BrdU incorporation in NP cells (Figures 7(c), 7(d), and 7(e), Supplementary Material, Figure S5). Furthermore, the upregulation of matrix proteases and proinflammatory mediators in the transfected cells was determined by RT-qPCR (Figures 7(f) and 7(g)).

### 3.5. The Involvement of Nox4 in IDD

**Figure 8:**
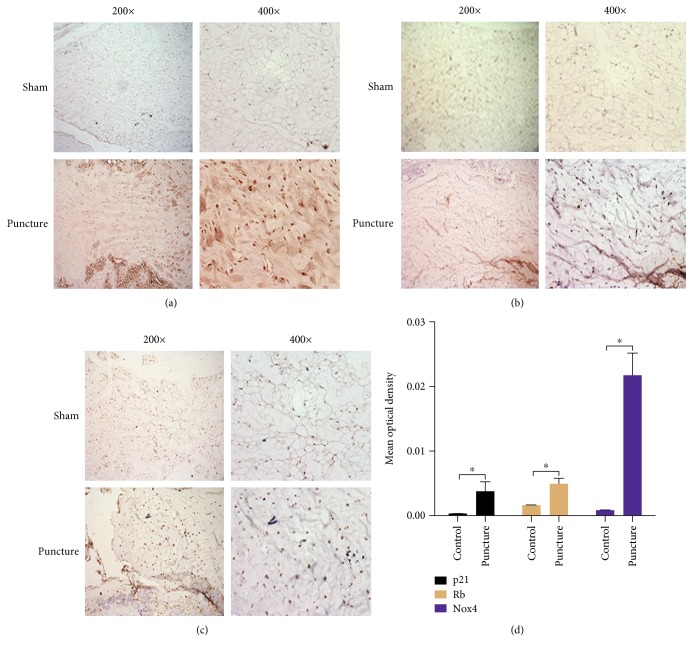
The expression of Nox4, p21, and Rb was upregulated in degenerative discs. (a) Immunohistochemical staining for Nox4 in NP specimens. (b) Immunohistochemical staining for p21 in NP specimens. (c) Immunohistochemical staining for Rb in NP specimens. (d) The mean optical density of Nox4, p21, and Rb in NP sections (*N* = 5). Sham: the sham control; puncture: the puncture discs. ^∗^, *P* value < 0.05, error bars represent standard error. 200x: 200 times magnification; 400x: 400 times magnification.

MRI and histological analysis demonstrated the degeneration of rat caudal discs induced by a puncture. The punctured discs showed lower T2 signal intensity, smaller NP area, disorganized AF, and enhanced connective tissue infiltration (Supplementary Material, Figure S6A, B). Notably, the MOD of Nox4 in the punctured NP was significantly higher than that in the sham group (Figures 8(a) and 8(d)). Also, the MOD of p21 and Rb markedly increased in the punctured NP (Figures 8(b), 8(c), and 8(d)).

## 4. Discussion

In the current study, we demonstrated that exposure of NP cells to high oxygen tension causes oxidative stress and DNA damage in NP cells. Consequently, high oxygen tension induced the cell cycle arrest of NP cells via the p53-p21-Rb and p16-Rb pathways. Also, it showed a catabolic and proinflammatory effect on the SASP of NP cells. These findings suggest that high oxygen tension is a potent trigger of disc cell senescence.

Noticeably, GSH and NAC significantly abrogated excessive ROS production in NP cells subjected to 20% O_2_. As a result, the DNA damage foci of NP cells were decreased, indicating the genotoxicity of ROS. However, the correlation between ROS and DNA damage is sophisticated. The involvement of DNA damage in ROS production of NP cells should be investigated in further studies. More importantly, the prosenescent of high oxygen tension on NP cells was prominently abolished by antioxidants, suggesting that ROS are critical mediators in high oxygen tension-induced premature senescence of NP cells. Interestingly, recent studies have been mentioned that oxidative stress essentially contributes to the establishment and progression of IDD [4, 19, 35]. Particularly, in aged or degenerative IVDs with neovascularization, disc cells inevitably were exposed to higher oxygen tension, leading to excessive ROS production that exerts detrimental effects on the viability and function of disc cells [14–16]. Undoubtedly, our study provides a strong support to this idea. High oxygen tension causes oxidative stress to suppress the proliferation of disc cells and to induce the expression of matrix protease and proinflammatory mediators by disc cells, which promotes the matrix remodeling and inflammation of degenerative discs. Thus, oxidative stress is a promising therapeutic target for IDD. Antioxidation is a potential approach for preventing disc cell senescence and disc degeneration.

MAPK and NF-*κ*B pathways are able to be activated by various stimuli in disc cells, including cytokines, growth factors, and high osmotic pressure. Consequently, physiological functions of disc cells are regulated to adapt to the stressful microenvironment [36, 37]. As mentioned above, NP cells developed senescent signs in response to high oxygen tension. Therefore, we were interested in revealing the roles of MAPK and NF-*κ*B pathways in high oxygen tension-induced premature senescence of NP cells. Herein, we found that the activation of both pathways induced by high oxygen tension was suppressed by antioxidants, indicating that the pathways are the downstream signaling of ROS in NP cells. More importantly, the different roles of MAPK and NF-*κ*B pathways in high oxygen tension-induced premature senescence of NP cells were determined using signaling inhibitors. On the one hand, the ERK inhibitor suppressed the expression of p53, p21, p16, and Rb in NP cells subjected to high oxygen tension, indicating that ERK activates the p53-p21-Rb and p16-Rb pathways to induce the cell cycle arrest of NP cells. On the other hand, the inhibitors of ERK, JNK, p38, and NF-*κ*B were shown to suppress the catabolic and proinflammatory effect of high oxygen tension on NP cells, suggesting that both MAPK and NF-*κ*B pathways are activated by ROS to regulate the catabolic and proinflammatory phenotype of NP cells. The involvement of MAPK and NF-*κ*B pathways in regulating disc cell viability and function in response to stimuli is required to be investigated in detail.

ROS originate from oxygen-using metabolic processes. Mitochondrion is a major site of ROS production in disc cells [16, 20]. A previous study has reported that increased oxygen tension (20% O_2_) significantly boosts mitochondrial-derived ROS production in NP cells. ROS disturbed matrix homeostasis of disc cells [16]. In consistent with the findings, we also found that 20% O_2_ prominently augmented ROS generation by NP cells. However, herein, we investigated nonmitochondrial ROS production through Nox4 in NP cells. Nox4 also is a major site of ROS production. Nox4-dependent ROS play crucial roles in regulating cell functions [26, 27]. Nevertheless, there were no studies that elucidate the roles of Nox in the ROS production and senescence of disc cells. In this study, high oxygen tension was found to markedly upregulate the expression of Nox4 in NP cells, suggesting that Nox4 is an oxygen-sensing enzyme and is regulated by oxygen tension at transcriptional level in NP cells. Furthermore, Nox4 knockdown abrogated the high oxygen tension-induced oxidative stress and DNA damage of NP cells. The activation of MAPK and NF-*κ*B pathways induced by high oxygen tension was also suppressed by siNox4. As a result, NP cell senescence induced by high oxygen tension was retarded, suggesting an essential contribution of Nox4 to high oxygen tension-induced senescence of NP cells. To determine the involvement of Nox4 in NP cell senescence further in depth, NP cells were transfected with Nox4 vectors for Nox4 overexpression. The cells overexpressing Nox4 produced excessive ROS and rapidly developed senescent signs, although at 1% O_2_, indicating that Nox4 is a major source of ROS in NP cells. The upregulation of Nox4 boots oxygen utilization to generate more genotoxic ROS to potently induce premature senescence of disc cells.

The rat caudal IDD model caused by needle puncture has been widely used [32, 33]. Therefore, we investigated the expression of Nox4 in degenerative discs *in vivo* using this model. As a result, the increased expression of Nox4 in degenerative NP tissues suggests the involvement of Nox4 in the pathogenesis of IDD. Furthermore, in consistent with Nox4, the expression of p21 and Rb in degenerative NP tissues also increased, providing a further *in vivo* evidence to support the association between Nox4 and NP cell senescence.

There are several limitations associated with our study. One limitation is that we investigated the effects of high oxygen tension and Nox4 on disc cell senescence based on rat disc cells. More studies based on human disc cells and IVD specimens should be performed to support our idea further. Secondly, although we discussed the involvement of Nox4-dependent ROS in NP cell senescence, the contribution of mitochondria-mediated ROS to disc cell senescence remains to be elucidated in the future. Besides, except for MAPK and NF-*κ*B pathways, more signaling pathways involved in regulating disc cell senescence should be discussed.

## 5. Conclusion

In summary, high oxygen tension markedly upregulates the expression of Nox4 to produce excessive ROS in NP cells. Oxygen-sensing Nox4-dependent ROS are genotoxic and activate the p53-p21-Rb and p16-Rb pathways via ERK signaling to induce cell cycle arrest of NP cells. Also, they induce the expression of matrix catabolic and proinflammatory genes in NP cells through MAPK and NF-*κ*B pathways. Moreover, with IDD progression, the expression of Nox4 is increased along with the upregulation of p21 and Rb in NP cells. Our study gives a novel insight into the causes and molecular mechanism of disc cell senescence, contributing to our understating of the pathogenesis of IDD. Ameliorating Nox4-dependent ROS production is not only a potential approach to retard disc cell senescence but also a promising therapeutic measure for IDD.

## Supplementary Material

The information of supplementary materials are as follows: Table 1. Abbreviations in this study. Figure S1. Senescence-associated β-galactosidase (SA-β-gal) staining of high oxygen tension-treated nucleus pulposus (NP) cells. NP cells were pretreated with glutathione (GSH) and N-acetyl-L-cysteine (NAC) for 30 min followed by high oxygen tension treatment for ROS scavenging. Figure S2. ERK inhibitor (U0126, U) suppressed the senescence-associated β-galactosidase (SA-β-gal) staining of high oxygen tension-treated nucleus pulposus (NP) cells. NP cells were pretreated with U for 30 min followed by high oxygen tension treatment. Figure S3. Small interfering RNA against Nox4 (siNox4) decreased the senescence-associated β-galactosidase (SA-β-gal) staining of high oxygen tension-treated nucleus pulposus (NP) cells. NP cells were transfected with siNox4 or scrambled siRNA control (siCtrl) before high oxygen tension treatment. Figure S4. Nucleus pulposus (NP) cells transfected with Nox4 vectors synthesized green fluorescent proteins (GFP). Figure S5. Nox4 overexpression reinforced the senescence-associated β-galactosidase (SA-β-gal) staining of nucleus pulposus (NP) cells. NP cells were transfected with Nox4 vectors for Nox4 overexpression. Figure S6. The rat caudal intervertebral disc degeneration caused by needle puncture. (A) Hematoxylin and eosin (H&E)-stained sections of rat caudal discs, 1 month after needle puncture. (B) MRI characteristics of the punctured level (C7-8) and the internal control levels (C6-7, C8-9), 1 month after needle puncture. Sham: the sham control. Puncture: the puncture discs. 40×: 40 times magnification; 100×: 100 times magnification; 200×: 200 times magnification. Figure S7. Adjusted relative expression fold of p53, p16, p21, Rb and p-Rb in NP cells. (A) NP cells were pretreated with GSH and NAC for 30 min followed by high oxygen tension treatment for ROS scavenging (N=5). (B) The adjusted relative expression fold of p-Rb was not affected by high oxygen tension (N=4). (C) NP cells were transfected with siNox4 before high oxygen tension treatment (N=3). (D) NP cells were transfected with Nox4 vectors for Nox4 overexpression (N=3). (E) NP cells were pretreated with GSH, NAC, the p38 inhibitor (SB202190, SB), the JNK inhibitor (SP600125, SP), the ERK inhibitor (U0126, U) or the NF-κB inhibitor (PDTC) for 30 min followed by high oxygen tension treatment for ROS scavenging or signaling inhibition (N=3). ∗, P value<0.05, error bars represent standard error. Figure S8. The phosphorylation level of ERK, JNK, p38, p65 in NP cells. (A) The phosphorylation of p38, ERK, JNK and p65 in NP cells treated with high oxygen tension for 15, 30, 45, 60 and 120 min. NP cells in 1% O_2_ served as the control (N=3). (B) NP cells were pretreated with GSH and NAC followed by high oxygen tension treatment for ROS scavenging (N=3). (C) NP cells were transfected with siNox4 before high oxygen tension treatment (N=3). ∗, P value<0.05, error bars represent standard error. Figure S9. Adjusted relative expression fold of Nox4 in NP cells. (A) NP cells were transfected with siNox4 or scrambled siRNA control (siCtrl) before high oxygen tension treatment (N=4). (B) NP cells were transfected with Nox4 vectors for Nox4 overexpression (N=3). ∗, P value<0.05, error bars represent standard error.

## Figures and Tables

**Table 1:** Primer sequences used in the real-time PCR analysis.

Target gene	Forward primer	Reverse primer
p53	GGGAATCTTCTGGGACGGGACA	CTGGTGGGCAGTGCTCTCTTTG
p21	CTGCCTGGTTCCTTGCCACTTC	GCTCTGGACGGTACGCTTAGGT
p16	CGTCGTGCGGTATTTGCGGTAT	GCGTTGCCAGAAGTGAAGCCA
Rb	AGCAGCCTCAGCCTTCCATACT	TGTTCTGGCTCTGGGTGGTCAG
MsrA	GGCAATGACTGTGGCACGCA	CCTCTCGGATGTCGGTGGTGAT
MsrB1	TCCTGTGGCAAGTGTGGCAATG	TGACTGAGGCTGGAGTGGTTGG
MsrB2	AGCAAGGCAGACTGGCAGAAGA	GGGCTATCACAGCACACGCAAT
Nox4	CGCACAGTCCTGGCTTACCTT	GGCAGCTACATGCACACCTGAG
MMP2	AGTGACGGCTTCCTCTGGTGTT	CAGGGCTGTCCATCTCCATTGC
MMP3	CCTGGCCCGTTTCCATCTCTCT	GGTGCTGACTGCATCGAAGGAC
MMP13	TACGAGCATCCATCCCGAGACC	TGAACCGCAGCACTGAGCCT
ADAMTS4	CCAGGACTTGTGGAGGTGGTGT	AAGAGGTCGGTTCGGTGGTTGT
ADAMTS5	GCTCCTCTTGGTGGCTGACTCT	GCGTTCTTGCTCACCTCCAGAC
IL6	AGCCCACCAGGAACGAAAGTCA	GGAAGGCAGTGGCTGTCAACAA
CCL2	CAGCCCAGAAACCAGCCAACTC	TGAGTAGCAGCAGGTGAGTGGG
CCL5	GACACCACTCCCTGCTGCTTTG	GCACACACTTGGCGGTTCCTT
CXCL10	TGTCGTTCTCTGCCTCGTGCT	CCTGGGTCTCAGCGTCTGTTCA
GAPDH	GTCCATGCCATCACTGCCACTC	GATGACCTTGCCCACAGCCTTG

Msr: methionine sulfoxide reductase; Nox: nicotinamide adenine dinucleotide phosphate oxidase; MMP: matrix metalloproteinase; ADAMTS: a disintegrin and metalloproteinase with thrombospondin motifs; IL: interleukin; CCL: C-C motif ligand; CXCL: C-X-C motif ligand; GAPDH: glyceraldehyde-3-phosphate dehydrogenase.
