# Supplementary material for: Oxygen-Sensing Nox4 Generates Genotoxic ROS to Induce Premature Senescence of Nucleus Pulposus Cells through MAPK and NF-κB Pathways
Source: Oxid Med Cell Longev. 2017 Sep 24;2017:7426458. doi: 10.1155/2017/7426458 (PMC5632907; doi:10.1155/2017/7426458)
Supplement: Supplementary file 1 — The information of supplementary materials are as follows: Table 1. Abbreviations in this study. Figure S1. Senescence-associated β-galactosidase (SA-β-gal) staining of high oxygen tension-treated nucleus pulposus (NP) cells. NP cells were pretreated with glutathione (GSH) and N-acetyl-L-cysteine (NAC) for 30 min followed by high oxygen tension treatment for ROS scavenging. Figure S2. ERK inhibitor (U0126, U) suppressed the senescence-associated β-galactosidase (SA-β-gal) staining of high oxygen tension-treated nucleus pulposus (NP) cells. NP cells were pretreated with U for 30 min followed by high oxygen tension treatment. Figure S3. Small interfering RNA against Nox4 (siNox4) decreased the senescence-associated β-galactosidase (SA-β-gal) staining of high oxygen tension-treated nucleus pulposus (NP) cells. NP cells were transfected with siNox4 or scrambled siRNA control (siCtrl) before high oxygen tension treatment. Figure S4. Nucleus pulposus (NP) cells transfected with Nox4 vectors synthesized green fluorescent proteins (GFP). Figure S5. Nox4 overexpression reinforced the senescence-associated β-galactosidase (SA-β-gal) staining of nucleus pulposus (NP) cells. NP cells were transfected with Nox4 vectors for Nox4 overexpression. Figure S6. The rat caudal intervertebral disc degeneration caused by needle puncture. (A) Hematoxylin and eosin (H&E)-stained sections of rat caudal discs, 1 month after needle puncture. (B) MRI characteristics of the punctured level (C7-8) and the internal control levels (C6-7, C8-9), 1 month after needle puncture. Sham: the sham control. Puncture: the puncture discs. 40×: 40 times magnification; 100×: 100 times magnification; 200×: 200 times magnification. Figure S7. Adjusted relative expression fold of p53, p16, p21, Rb and p-Rb in NP cells. (A) NP cells were pretreated with GSH and NAC for 30 min followed by high oxygen tension treatment for ROS scavenging (N=5). (B) The adjusted relative expression fold of p-Rb was not affected by hig [file 7426458.f1.pdf]

Table 1. Abbreviations in this study

IVD	intervertebral disc
IDD	intervertebral disc degeneration
SASP	senescence-associated secreted phenotype
NP	nucleus pulposus
AF	annulus fibrosus
SA-β-Gal	senescence-associated β-galactosidase
ECM	extracellular matrix
Nox4	NADPH oxidase 4
GSH	glutathione
Rb	retinoblastoma protein
NAC	N-acetyl-L-cysteine
ADAMTS	a disintegrin and metalloproteinase with thrombospondin motifs
MMP	matrix metalloproteinase
p-Rb	phosphorylated retinoblastoma protein
ROS	reactive oxygen species
U	U0126
SB	SB202190
FBS	fetal bovine serum
SP	SP600125

PBS	phosphate-buffered saline
siRNA	small interfering RNA
GFP	green fluorescent protein
MOD	mean optical density
Msr	methionine sulfoxide reductase
IL	interleukin
CCL	C-C motif ligand
CXCL	C-X-C motif ligand
SIPS	stress-induced premature senescence

Figures

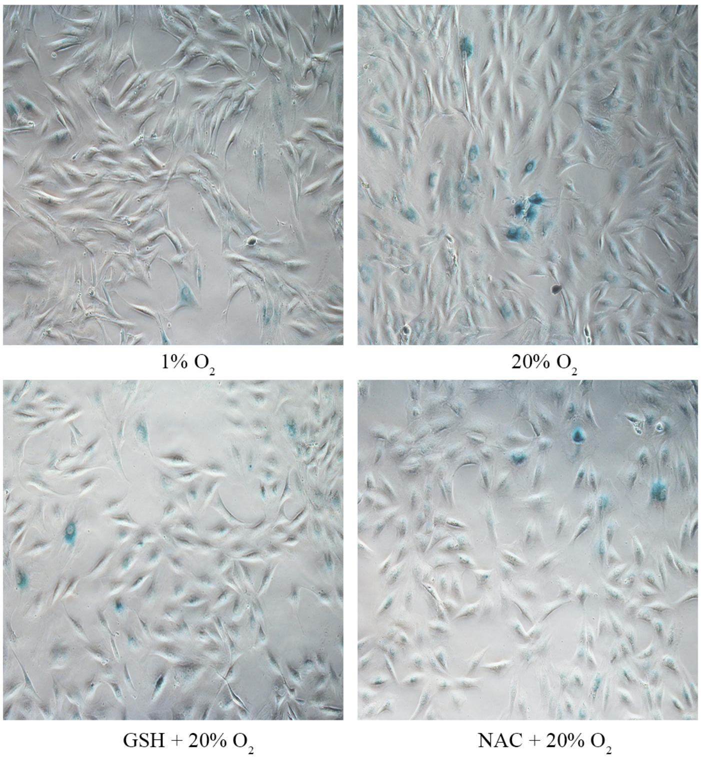

Figure S1. Senescence-associated β-galactosidase (SA-β-gal) staining of high oxygen tension-treated nucleus pulposus (NP) cells. NP cells were pretreated with glutathione (GSH) and N-acetyl-L-cysteine (NAC) for 30 min followed by high oxygen tension treatment for ROS scavenging.

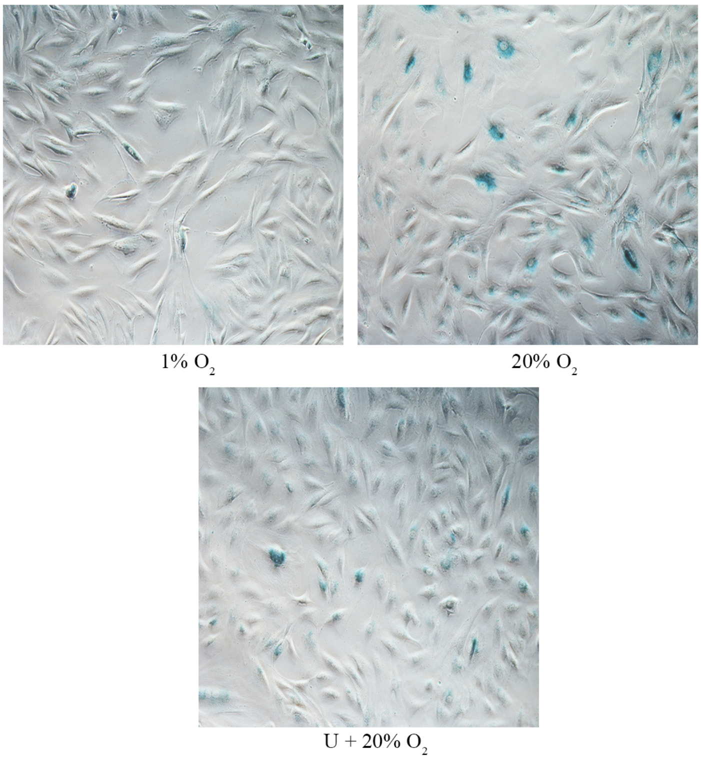

Figure S2. ERK inhibitor (U0126, U) suppressed the senescence-associated β-galactosidase (SA-β-gal) staining of high oxygen tension-treated nucleus pulposus (NP) cells. NP cells were pretreated with U for 30 min followed by high oxygen tension treatment.

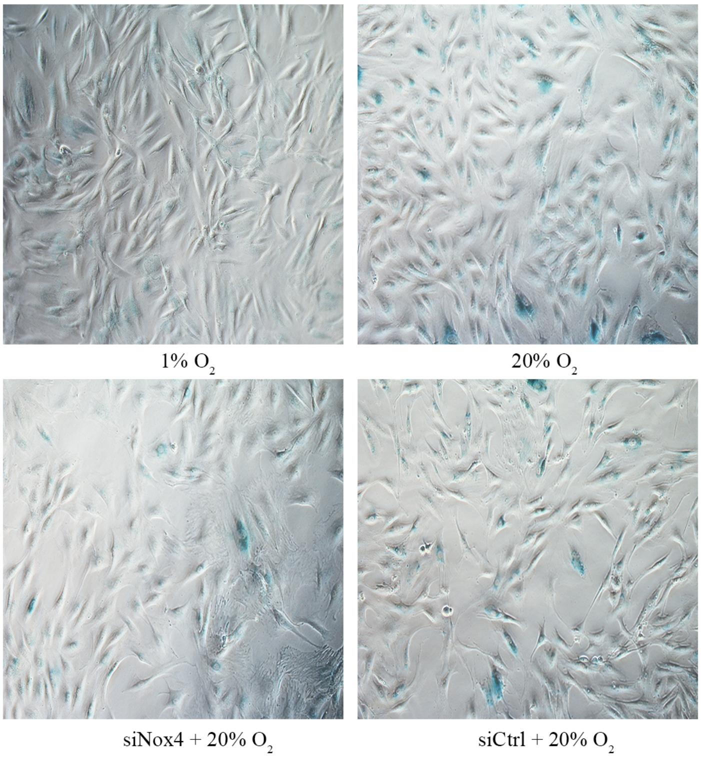

Figure S3. Small interfering RNA against Nox4 (siNox4) decreased the senescence-associated β-galactosidase (SA-β-gal) staining of high oxygen tension-treated nucleus pulposus (NP) cells. NP cells were transfected with siNox4 or scrambled siRNA control (siCtrl) before high oxygen tension treatment.

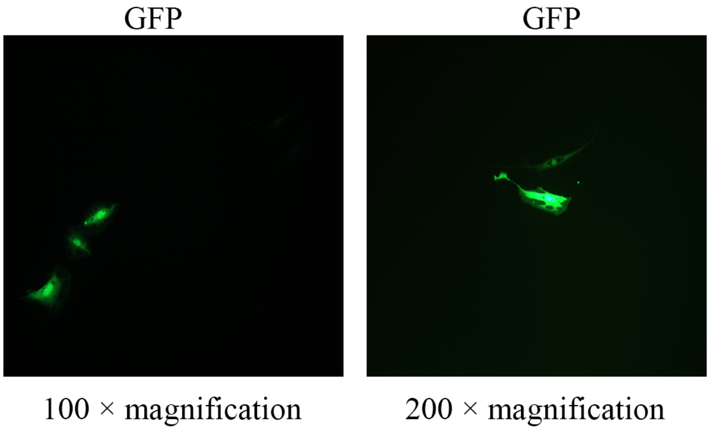

Figure S4. Nucleus pulposus (NP) cells transfected with Nox4 vectors synthesized green fluorescent proteins (GFP).

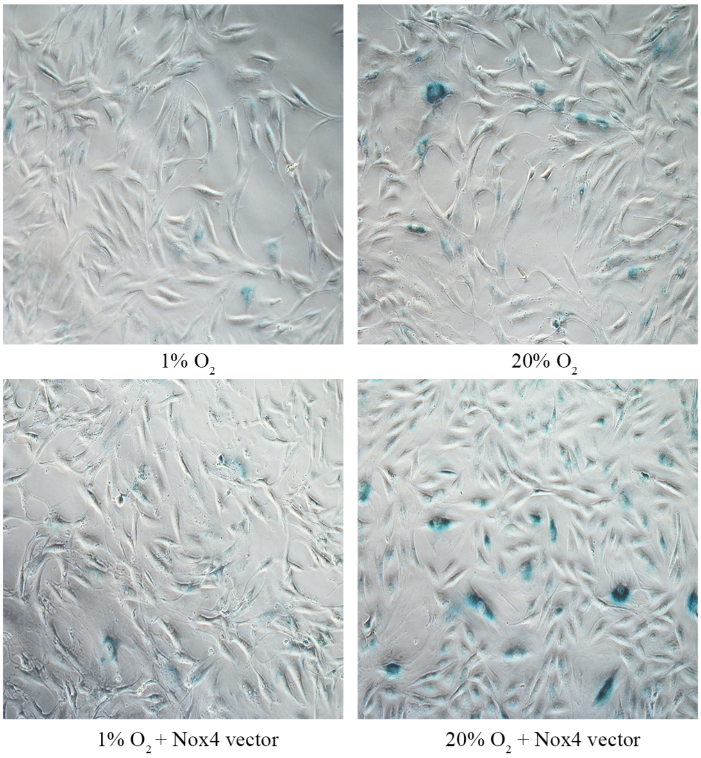

Figure S5. Nox4 overexpression reinforced the senescence-associated β-galactosidase (SA-β-gal) staining of nucleus pulposus (NP) cells. NP cells were transfected with Nox4 vectors for Nox4 overexpression.

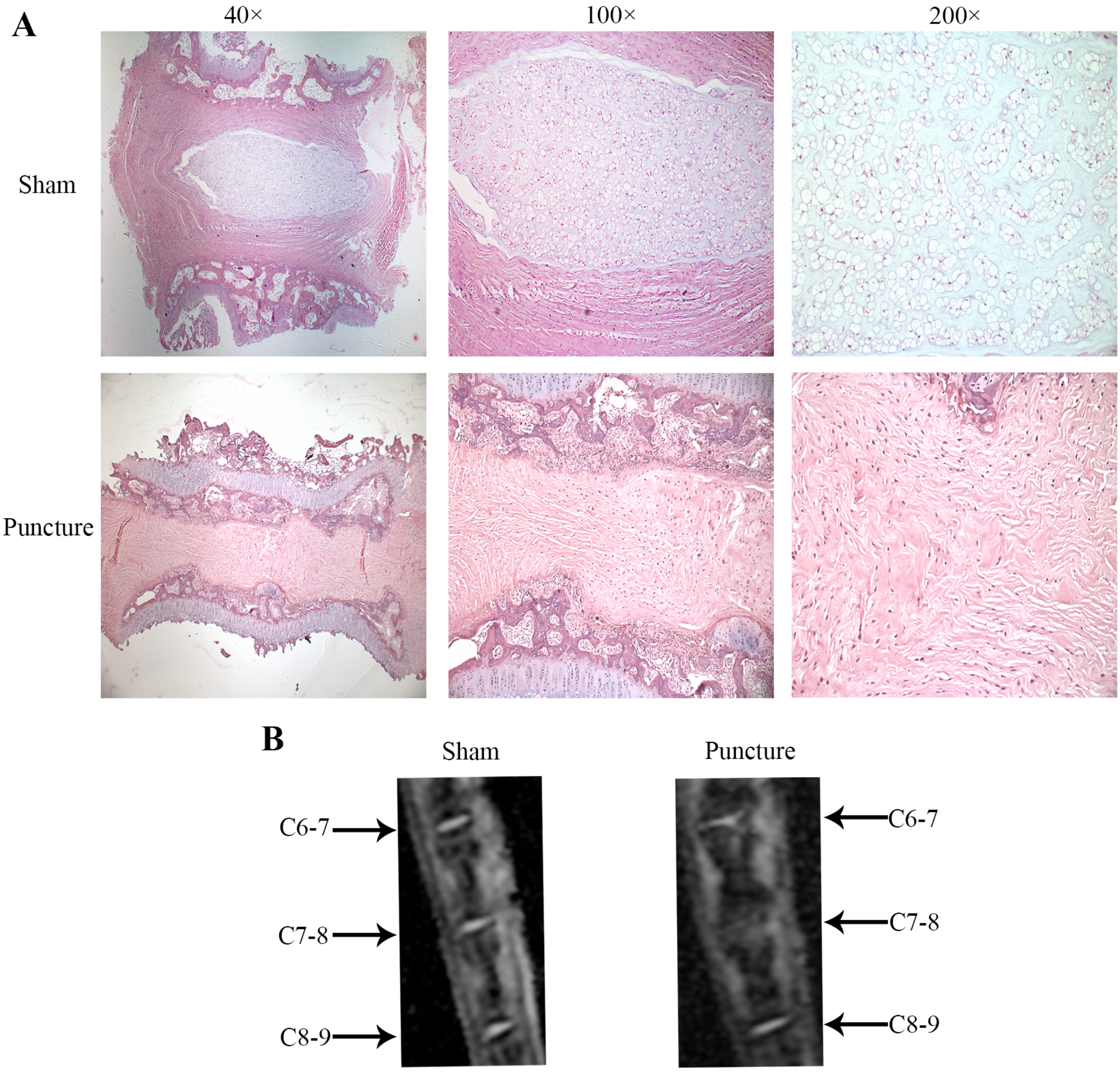

Figure S6. The rat caudal intervertebral disc degeneration caused by needle puncture. (A) Hematoxylin and eosin (H&E)-stained sections of rat caudal discs, 1 month after needle puncture. (B) MRI characteristics of the punctured level (C7-8) and the internal control levels (C6-7, C8-9), 1 month after needle puncture. Sham: the sham control. Puncture: the puncture discs. 40×: 40 times magnification; 100×: 100 times magnification; 200×: 200 times magnification.

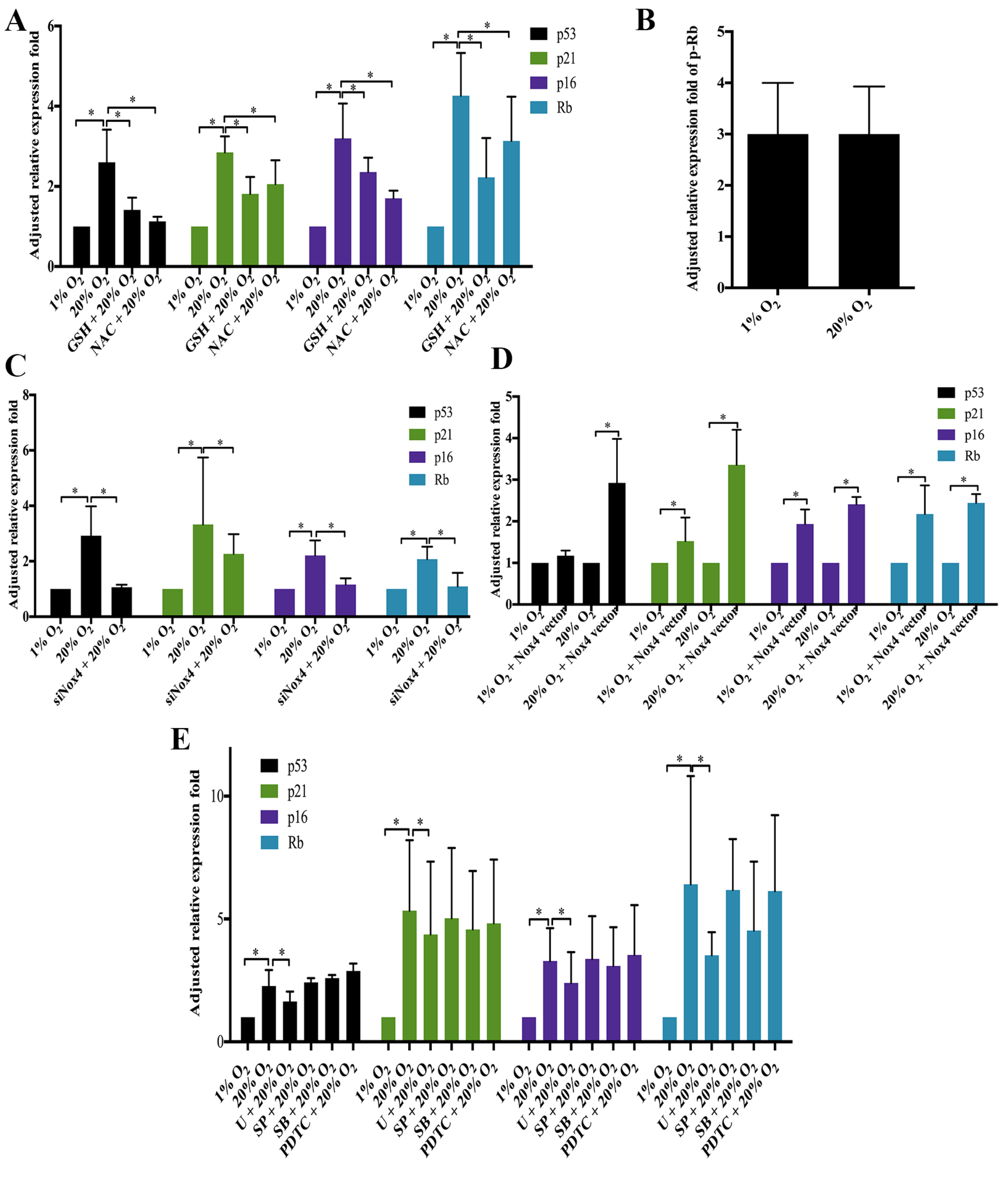

Figure S7. Adjusted relative expression fold of p53, p16, p21, Rb and p-Rb in NP cells. (**A**) NP cells were pretreated with GSH and NAC for 30 min followed by high oxygen tension treatment for ROS scavenging (N=5). (**B**) The adjusted relative expression fold of p-Rb was not affected by high oxygen tension (N=4). (**C**) NP cells were transfected with siNox4 before high oxygen tension treatment (N=3). (**D**) NP

cells were transfected with Nox4 vectors for Nox4 overexpression (N=3). (**E**) NP cells were pretreated with GSH, NAC, the p38 inhibitor (SB202190, SB), the JNK inhibitor (SP600125, SP), the ERK inhibitor (U0126, U) or the NF-κB inhibitor (PDTC) for 30 min followed by high oxygen tension treatment for ROS scavenging or signaling inhibition (N=3). *, P value<0.05, error bars represent standard error.

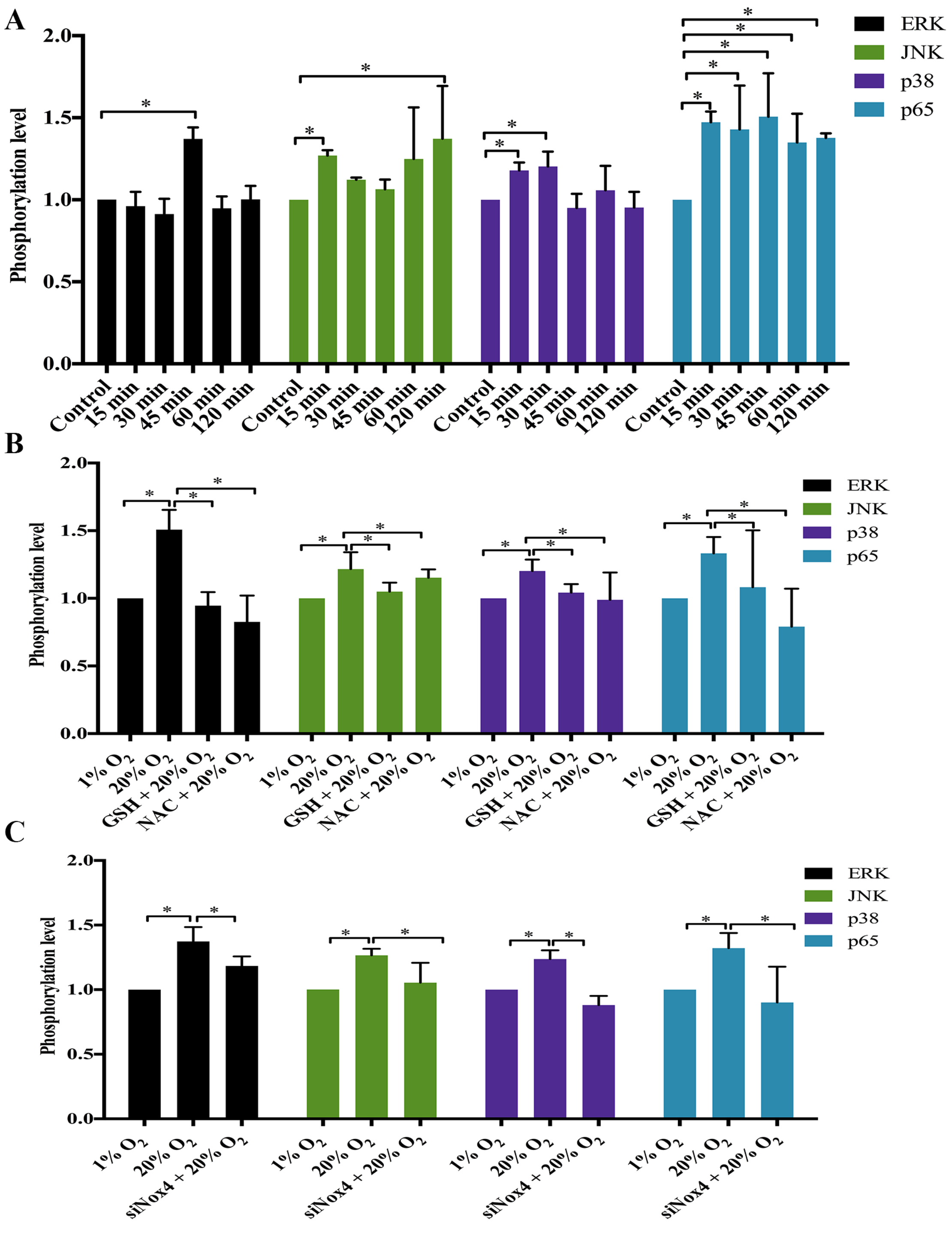

Figure S8. The phosphorylation level of ERK, JNK, p38, p65 in NP cells. (**A**) The phosphorylation of p38, ERK, JNK and p65 in NP cells treated with high oxygen tension for 15, 30, 45, 60 and 120 min. NP cells in 1% O_2 served as the control (N=3). (**B**) NP cells were pretreated with GSH and NAC followed by high oxygen tension treatment for ROS scavenging (N=3). (**C**) NP cells were transfected with siNox4

before high oxygen tension treatment (N=3). *, P value<0.05, error bars represent standard error.

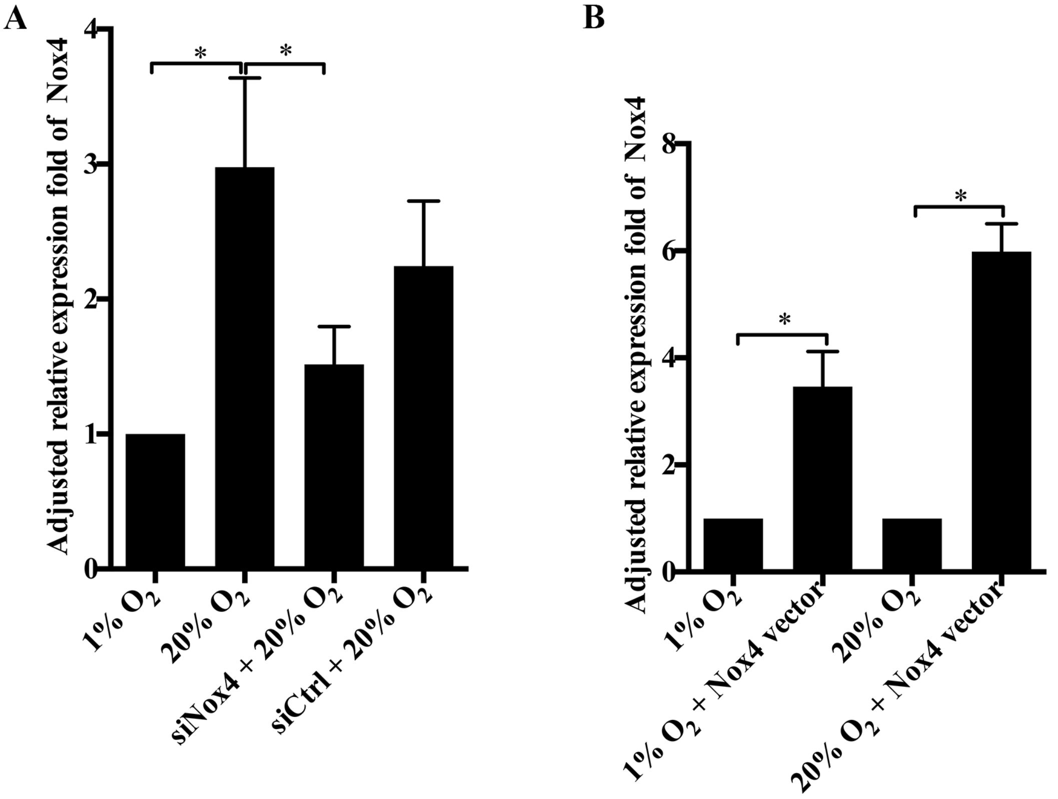

Figure S9. Adjusted relative expression fold of Nox4 in NP cells. (**A**) NP cells were transfected with siNox4 or scrambled siRNA control (siCtrl) before high oxygen tension treatment (N=4). (**B**) NP cells were transfected with Nox4 vectors for Nox4 overexpression (N=3). *, P value<0.05, error bars represent standard error.
