# Supplementary material for: Oxygen-Sensing Nox4 Generates Genotoxic ROS to Induce Premature Senescence of Nucleus Pulposus Cells through MAPK and NF-κB Pathways
Source: Oxid Med Cell Longev. 2017 Sep 24;2017:7426458. doi: 10.1155/2017/7426458 (PMC5632907; doi:10.1155/2017/7426458)
Supplement: Supplementary file 2 [file 7426458.f2.docx]

| Table 1. Abbreviations in this study | |  |
| --- | --- | --- |
| IVD | intervertebral disc | |
| IDD | intervertebral disc degeneration | |
| SASP | senescence-associated secreted phenotype | |
| NP | nucleus pulposus | |
| AF | annulus fibrosus | |
| SA-β-Gal | senescence-associated β-galactosidase | |
| ECM | extracellular matrix | |
| Nox4 | NADPH oxidase 4 | |
| GSH | glutathione | |
| Rb | retinoblastoma protein | |
| NAC | Ν-acetyl-L-cysteine | |
| ADAMTS | a disintegrin and metalloproteinase with thrombospondin motifs | |
| MMP | matrix metalloproteinase | |
| p-Rb | phosphorylated retinoblastoma protein | |
| ROS | reactive oxygen species | |
| U | U0126 | |
| SB | SB202190 | |
| FBS | fetal bovine serum | |
| SP | SP600125 | |
| PBS | phosphate-buffered saline | |
| siRNA | small interfering RNA | |
| GFP | green fluorescent protein | |
| MOD | mean optical density | |
| Msr | methionine sulfoxide reductase | |
| IL | interleukin | |
| CCL | C-C motif ligand | |
| CXCL | C-X-C motif ligand | |
| SIPS | stress-induced premature senescence | |
